# Antitumor effects of polysaccharides from *Tetrastigma hemsleyanum* Diels et Gilg *via* regulation of intestinal flora and enhancing immunomodulatory effects *in vivo*

**DOI:** 10.3389/fimmu.2022.1009530

**Published:** 2022-10-28

**Authors:** Fangmei Zhou, Yan Lu, Tong Sun, Ling Sun, Bixu Wang, Jingjing Lu, Zhimin Li, Bingqi Zhu, Shigao Huang, Zhishan Ding

**Affiliations:** ^1^ School of Medical Technology and Information Engineering, Zhejiang Chinese Medical University, Hangzhou, China; ^2^ First College of Clinical Medicine, Zhejiang Chinese Medical University, Hangzhou, China; ^3^ Information Technology Center, Zhejiang Chinese Medical University, Hangzhou, China; ^4^ Department of Radiation Oncology, The First Affiliated Hospital, Air Force Medical University, Xi an, China

**Keywords:** *tetrastigma hemsleyanum*, polysaccharides, antitumor activity, immunomodulation, cyclophosphamide, intestine

## Abstract

*Tetrastigma hemsleyanum* Diels et Gilg is a traditional Chinese herbal medicine with high medicinal value, and antitumor, antioxidant and anti-inflammatory biological activities. However, while several studies have focused on flavonoids in *Tetrastigma hemsleyanum* tubers, there are few studies on the enhanced immune effect of *Tetrastigma hemsleyanum* polysaccharides (THP). In this study, we evaluated the antitumor effect of THP in a lung tumor model and explored the mechanism of antitumor activity through intestinal flora. In addition, a cyclophosphamide (CTX)-induced immunosuppression model was used to declare the immunomodulatory effect of THP in the immunosuppressive state induced by antitumor drugs. The results showed that THP increased the content of ileum secreted immunoglobulin A (SIgA) and cecum short-chain fatty acids (SCFAs) and improved microbial community diversity, regulating the relative abundance of dominant microbiota flora from the phylum level to the genus level, and recovering the intestinal microflora disorder caused by tumors. Additionally, THP can increase the organ indices and improve immune organ atrophy. THP can upregulate routine blood counts and stimulate the production of the serum cytokines. THP also promoted the macrophage phagocytic index, NK-cell activation, and complement and immunoglobulin (IgG, IgA, IgM) levels. The detection of Splenic lymphocyte proliferation and T lymphocyte subsets also sideways reflects that THP can restore CTX-induced immune inhibition in mice. In conclusion, this study suggests that THP can effectively achieve the enhanced antitumor effects, regulate gut microbiota and improve the immunosuppression induced by antitumor drugs. Therefore, THP can enhance the immune capacity and provide novel immunomodulatory and antineoplastic adjuvant agents.

## Graphical Abstract

Mechanism of the antitumor and immunomodulatory action of THP. THP exerts an antitumor role by regulating intestinal flora. Meanwhile, THP plays an immunomodulatory role through specific immunity, nonspecific immunity and the intestinal tract. Created with BioRender.com.

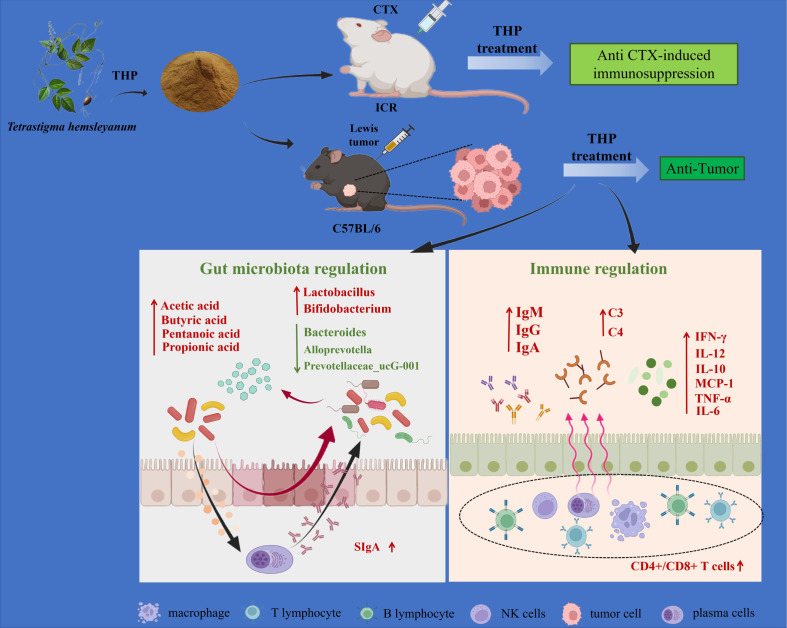

## 1 Introduction

Cancer causes numerous deaths worldwide (1), and lung cancer is the leading cause of cancer death worldwide (2), mainly because it is initially asymptomatic and is usually detected at a later stage (3). Comprehensive therapies such as local treatment and chemoradiotherapy (4) are mainly used for the clinical treatment of lung cancer at present, and novel therapies such as immune checkpoint inhibitors and tyrosine kinase inhibitors are emerging, which have achieved good curative effects (5, 6). In particular, CTX is the preferred drug for cancer treatment and can be used in various types of cancer therapy (7). Regrettably, excessive use of CTX will damage the immune system and reduce the immune function of the body (8), resulting in a series of side effects such as oxidative stress and immunosuppression (9). Immune homeostasis is crucial to human health. Under normal physiological conditions, the immune system can defend against external invasion and protect human health (10). After the immune system is damaged, the body becomes more sensitive to pathogens and is inevitably more vulnerable to viral attacks, resulting in or aggravating various diseases, especially cancer. Therefore, the grim reality compels us to find a highly effective and low-toxicity substance to treat cancer.

In recent years, numerous studies (11, 12) have shown that traditional Chinese medicine (TCM) has good antitumor activity, multiple components, and targets in cancer treatment, which will lead to good efficacy and side effects. It can improve the immunity of the body and delay the progress of the disease, showing unique advantages in the comprehensive treatment of tumors (13). Polysaccharides are a kind of natural biological macromolecule substance that are an indispensable part of the body (14). To date, researchers have isolated a certain amount of polysaccharides, which are widely distributed in plants, animals, and microorganisms (15), and have been found to have various biological activities, such as immunomodulatory, antitumor, anti-inflammatory, and antioxidation activities (16, 17). Polysaccharides have fewer side effects on the human body, and their immunomodulatory and antitumor effects have become a hot spot of biomedical and biological science research (18). Most antitumor polysaccharides can inhibit the proliferation of tumor cells, induce the activation of dendritic cells and promote the proliferation of immune cells (19, 20), and some can directly kill tumor cells (21) or induce apoptosis of tumor cells (17). In addition, some polysaccharides can activate macrophages and lymphocytes (including NK cells, T cells and B cells) to enhance the body’s immune function (22, 23). Therefore, it is very meaningful to explore the antitumor and immunomodulatory effects of polysaccharides from TCM.

*Tetrastigma hemsleyanum* (TH), belongs to rhamniaceae and vines of the grapevine family. It is a unique traditional medicinal plant in China. Both the root tuber and whole grass of TH have been used as folk medicines with a long history, and their active ingredients include flavonoids, organic acids, polysaccharides, etc. A large number of ancient Chinese books record that TH has the effect of clearing heat and promoting blood circulation and can treat diseases such as high fever and pneumonia (24). In our previous study, we confirmed the antitumor activity of the TH, however, the mechanism remained unclear (25, 26). Encouragingly, polysaccharides are one of the main active components of TH. In previous studies, it was found that *Tetrastigma hemsleyanum* polysaccharide (THP) has various biological activities (27). However, regrettably, there have been few studies on THP’s immunomodulatory and antitumor mechanismst. On this basis, if we can prove that TH has therapeutic effects on immunosuppression side effects caused by chemotherapy, TH can exert more powerful therapeutic effects through antitumor and immunomodulatory aspects, and it may become an important antitumor adjunct drug.

The intestinal flora is a complex microecosystem in the human body, and the occurrence of some diseases is closely related to its imbalance (28). In recent years, experiments have revealed that gut microflora dysbiosis can accelerate the progression of colorectal cancer (29), breast cancer (30), lung disease (31), etc. Some of the intestinal flora that are endowed with cancer susceptibility may contribute to the occurrence and development of tumors to a certain extent (32). It was found in a study of COVID-19 that SARS-CoV-2 infection in primates was also associated with altered intestinal flora composition and functional activity (33). Thus, dysregulation of the microbiome has been related to systemic inflammatory disease (34, 35) and various types of cancer (36). In addition, intestinal flora also influence the effectiveness of chemotherapy in cancer patients (37, 38), which means maintaining a good intestinal flora plays an important role in the treatment of diseases (39). In recent years, several studies have shown that polysaccharides can dynamically regulate the intestinal microenvironment according to intestinal flora and mucosal immunity, and regulate the development and process of cancer (40, 41). Therefore, it is necessary to explore the relationship between the antitumor effect of THP and intestinal flora.

Our previous studies have identified the structure of THP and demonstrated that THP can have an antitumor effect on Lewis tumor-bearing mice (26), but the antitumor mechanism of THP in Lewis tumor bearing mice is still unclear. Therefore,a Lewis lung cancer-bearing mouse model was established to investigate the effect of THP and the relationship between its antitumor effects and intestinal flora, which would be beneficial for the prevention and therapy of lung cancer. In addition, to explore the immunomodulatory effect of THP on CTX immunosuppressed mice, CTX immunosuppressed mice were established to detect mucosal immunity, innate immunity, cellular immunity and humoral immunity. These results provide a novel theoretical basis for THP as an adjuvant chemotherapy agent in cancer treatment.

## 2 Materials and methods

### 2.1 Materials and chemicals

The aerial parts of *Tetrastigma hemsleyanum* Diels et Gilg were collected from Hangzhou Sanri Agri-Tech Co., Ltd. (Hangzhou, China), which were provided by Xindeng Planting Base, Zhejiang Province, China, in March 2019. The plant was authenticated by Professor Ding Zhishan of Zhejiang Chinese Medical University. The voucher specimen was stored in the School of Medical Technology and Information Engineering, Zhejiang Chinese Medicine University, China.

Lewis cells and YAC-1 cells were purchased from the Cell Bank of Chinese Academy of Science (Shanghai, China). Acetic acid, propionic acid, butyric acid and pentanoic acid were purchased from Sinopharm Chemical Reagent Co., Ltd. (Zhejiang, China). Cyclophosphamide (CTX) was purchased from Shanghai YuanYe Bio-Technology Co., Ltd. (Shanghai, China). Ampicillin sodium, vancomycin hydrochloride, metronidazole and neomycin were obtained from Hangzhou Hechi Chemical Co., Ltd. (Hangzhou, China). A Cytometric Bead Array (CBA) Mouse Inflammation Kit was purchased from Bio Union Supply Chain Management Co., Ltd. (Beijing,China). Lipopolysaccharide (LPS) and concanavalin A (Con A) were purchased from Sigma Chemical Co. (MO, United States). India ink was purchased from Fuzhou Phygene Biotechnology Co., Ltd (Fuzhou, China). HE dye was purchased from Zhuhai Baso Biotechnology Co., Ltd (Zhuhai, China). All other chemicals were reagent grade from Sinopharm Chemical Reagent Co. Ltd. (Shanghai, China) and Merck (Darmstadt, Germany). The water used in this study was deionized water obtained from a Merck Milli-Q water purification system (Darmstadt, Germany).

### 2.2 Extraction and purification of THP

THP was prepared in our laboratory as previously described (42). The dried aerial parts of *Tetrastigma hemsleyanum* were powdered with a blender. The plant powder was extracted with distilled water for 90 min, filtered for centrifugation, and concentrated under vacuum. The concentrated water extract was added to a triple volume of 95% ethanol solution, incubated and precipitated for 12 h at 4 °C. The precipitate was lyophilized to obtain a crude polysaccharide. The Sevaga method was applied to deproteinated crude polysaccharides. The deprotein samples were purified *via* a DEAE-Sepharose fast column and Superdex-20 0 chromatography column to obtain purified polysaccharides, which were named THP. The molecular weight was determined on a Water Ultrahydrogel 500 Column (Milford, MA, USA) by high-performance gel penetration chromatography. The contents of neutral sugar and uronic acid were determined by phenol-sulfuric acid colorimetry, and the composition of monosaccharides was analyzed by the Blumenkrantz and Asboehansen methods. The monosaccharide composition of THP was determined by gas chromatography-mass spectrometry, and analyzed by infrared spectroscopy on a Nicolet IS5 Fourier transform infrared (FTIR) spectrometer (Waltham, MA, USA).

### 2.3 Cytotoxicity evaluation by MTT assay

The MTT method was employed to determine the effects of THP on lung cancer cells (Lewis) and RAW264.7 cells. Briefly, cells were seeded in a 96-well plate containing 5 × 10^4^ cells/well for 12 h and then treatment with THP at various concentrations (0.1, 1, 10, 100 and 1000 μg/ml) for 24 h. After incubation, 20 μL MTT solution (final concentration of 0.5 mg/mL) was added to each well. After further incubation for 4h, the supernatant was removed and 150 μL DMSO was subsequently added to each well. The absorbance at 570 nm wasmeasured with a microplate reader. Cell viability (%) = [(OD_treatment_ − OD_blank_)/(OD_control_ − OD_blank_)] × 100%

### 2.4 *In vivo* animal model

#### 2.4.1 Experimental animals

All experiments were conducted following the National Institutes of Health (NIH) Guide for the Care and Use of Laboratory Animals and approved by the Animal Subjects Review Board of Zhejiang Chinese Medical University(Hangzhou, China, Approval Number: SYXK(ZHE) 2018-0012). Healthy male ICR mice (five weeks old, body weight 20-24 g) and SPF male C57BL/6 mice (five weeks old, body weight 18-22 g) were obtained from the Experimental Animal Center of Zhejiang Chinese Medicine University (the license number: SYXK(Zhe)2018-0012). All animals were housed in standard conditions (25 ± 3 °C, 60 ± 5% humidity and a 12 h light/dark cycle) with free access to complete pellet feed and water. All animals were acclimated for seven days before the experiment.

#### 2.4.2 Lewis tumor-bearing mice

**Figure 1:**
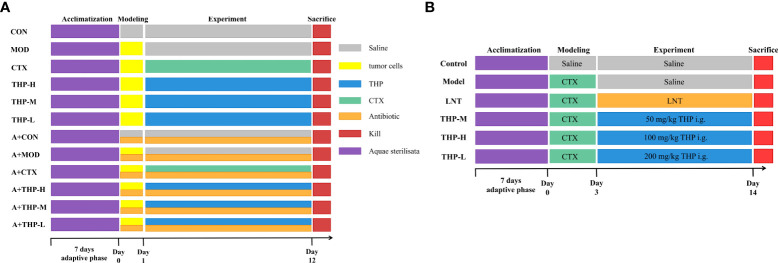
Grouping and administration of mice. **(A)** The antitumor lung cancer model animal experimental protocol. **(B)** The immunosuppression model animal experimental protocol. Control, normal control group; Model, model control group; LNT, Lentinan group; THP-L, THP low dose group (50 mg/kg); THP-M, THP medium dose group (100 mg/kg); THP-H, THP high dose group (200 mg/kg); CON, normal control group; MOD, tumor model control group; CTX, CTX group; THP-L, THP low dose group (50 mg/kg); THP-M,THP medium dose group (150 mg/kg); THP-H, THP high dose group (200 mg/kg); A+CON, antibiotic+normal control group; A+MOD, antibiotic+tumor model control group; A+CTX, antibiotic+CTX group; A+THP-L, antibiotic+THP low dose group (50 mg/kg); A+THP-M, antibiotic+THP medium dose group (150 mg/kg); A+THP-H, antibiotic+THP high dose group (200 mg/kg).

As shown in **Figure 1A**. All C57BL/6 mice were randomly divided into twelve groups (10 mice for each group): (1) normal control group (CON), (2) tumor model group (MOD), (3) CTX group (CTX), (4) THP low-dose group (THP-L), (5) THP medium-dose group (THP-M), (6) THP high-dose group (THP-H), (7) antibiotic+normal control group (A+CON), (8) antibiotic+tumor model control group (A+MOD), (9) antibiotic+CTX group (A+CTX), (10) antibiotic+THP low-dose group (A+THP-L), (11) antibiotic+THP medium dose-group (A+THP-M), and (12) antibiotic+THP high-dose group (A+THP-H). Each mouse was subcutaneously inoculated with Lewis lung cancer tumor cell suspension in the armpit of the right upper except, for the CON group. Mice in the CON and MOD groups were intragastrically administered saline once a day. The CTX group was intraperitoneally injected with 50 mg/kg CTX every other day, and the THP-L, THP-M and THP-H groups were intragastrically administered 50, 150 and 200 mg/kg THP every day, respectively. Groups (7) ~ (12) were simultaneously treated with an antibiotic cocktail (vancomycin 0.1 g/L, ampicillin 0.2 g/L, neomycin 0.2 g/L, metronidazole 0.2 g/L) to establish an intestinal disease model. The drug was administered continuously for 12 days.

#### 2.4.3 CTX immunosuppressed mice

As shown in **Figure 1B**. Male ICR mice were randomly divided into a normal control group (control), model control group (model), positive control group (LNT) and THP low-dose (THP-L), medium-dose (THP-M) and high-dose groups (THP-H), with 15 mice in each group. Except for the control group, the other groups were injected intraperitoneally with CTX 80 mg·kg^-1^, once a day, for 3 consecutive days to establish the immunosuppressive mouse model. CTX for injection should be prepared with sterile normal saline at an appropriate concentration [80 mg·kg^-1^)]. After establishing the animal model, the control group and model group were given normal saline by gavage. In the positive control group, lentinan was gavaged, and the THP of the low-, medium- and high-dose groups were intragastrically administered 50, 100 and 200 mg/kg THP every day, respectively. Intraperitoneal injection (divided into three times) and gavage were carried out at the same time in the morning every day for 1-3 days, and only gavage was needed for 4-15 days.

### 2.5 Antitumor effect of THP in Lewis tumor-bearing mice

#### 2.5.1 Tumor inhibition rate

After the last administration, the mice were fasted for 12 h, and all of the experimental mice were weighed. The tumor was carefully removed and weighed immediately. The tumor inhibition rate was calculated as follows:


Tumor inhibition rate(%)=(M1−M2)/M1×100%

Note: M1, the average tumor weights of the tumor model group; M2, the average tumor weights of the treated group.

Tumor tissue was fixed with 4% paraformaldehyde, then dehydrated, embedded, sectioned and stained with HE. The status of tissue necrosis was observed under a microscope(Eclipse TS100; Nikon Corporation, Tokyo, Japan).TUNEL (TdT-mediated dUTP Nick-End Labeling) assay was performed to detect apoptosis of tumor cells, using the TUNEL Apoptosis Assay Kit (Beyotime Institute of Biotechnology) according to the manufacturer’s instructions. The results was observed under a fluorescence microscope (TI, Nikon Corporation, Tokyo, Japan) at × 200 magnification.

#### 2.5.2 Fecal DNA extraction and 16S rRNA gene sequencing

Microbial community genomic DNA was extracted from fecal samples of the mice by using the E.Z.N.A.^®^ stool DNA Kit (Omega Bio-Tek, Norcross, GA, U.S.) according to the manufacturer’s instructions. The extraction quality of the DNA was detected by 1% agarose gel electrophoresis, and the concentration and purity of DNA were determined by a NanoDrop 2000 UV-vis spectrophotometer (Thermo Scientific, Wilmington, USA). PCR amplification of the V3-V4 variable region of the 16S rRNA gene was performed using 338F (5’-ACTCCTACGGGAGCAGCAG-3’) and 806R (5’-GGACTACHVGGGTWTCTAAT-3’) using an ABI GeneAmp^®^ 9700 PCR thermocycler (ABI, CA, USA). PCR products were extracted from a 2% agarose gel and purified using the AxyPrep DNA Gel Extraction Kit (Axygen Biosciences, Union City, CA, USA) according to the manufacturer’s instructions, and a quantus fluorimeter (Promega, USA) was used for quantification. The sequencing library was built using a NextFlex Rapid DNA-Seq Kit (Illumina, SD, USA) and then sequenced using an Illumina MiSeq PE300 platform (Majorbio Bio-Pharm Technology Co. Ltd). Operational taxonomic units (OTUs) with a 97% similarity cutoff were clustered using UPARSE (version 7.1, http://drive5.com/uparse/), and chimeric sequences were identified and removed. The taxonomy of each OTU representative sequence was analyzed by RDP Classifier (http://rdp.cme.msu.edu/) against the 16S rRNA database (e.g. Silva v138) using a confidence threshold of 0.7. The raw reads were deposited into the NCBI Sequence Read Archive (SRA) database (Accession Number: SRP379235).

### 2.6 Analysis of immunoglobulin (IgG, IgA, IgM) and SIgA

The levels of IgG, IgA and IgM in mouse serum were measured by ELISA kits (Nanjing, China) according to the mamusfacturer’s instructions. The abundance of SIgA in of the ileum was determined using ELISA kits (Yancheng, China) according to the manufacturer’s instructions.

### 2.7 Determination of SCFAs content

The levels of SCFAs (acetic acid, propionic acid, butyric acid and pentanoic acid) were determined by a Trace 1300 gas chromatograph (GC) and flame ionization detector (FID). Briefly, the cecal contents (100 mg) were mixed with 500 μL saturated NaCl solution for 30 min and then 100 μL 10% H_2_SO_4_ and 800 μL ether were added, the supernatant was collected after shaking and centrifugation at 12000 r/min for 15 min. The operating conditions were set as follows: the injection temperature and detector temperature were 200 °C and 250 °C, respectively, and the flow rate was 1 mL/min. The column temperature was 100 °C, when the sample was injected, which was kept for 2 min, then increased to 200 °C at 8 °C/min for 2 min, and the total running time was 16.5 min.

### 2.8 Analysis of the content of cytokine

The levels of IL-2, TNF-α and INF-γ in Lewis tumor-bearing mice serum were measured by ELISA Kits (Nanjing jiancheng, China) according to the manufacturer’s instructions.

The contents of IFN-γ, IL-10, TNF-α, IL-12, IL-6 and MCP-1 in CTX immunosuppressed mice serum were detected using a Cytometric Bead Array Mouse Th1/Th2/Th17 Cytokine Kit (BD Biosciences, San Diego, United States) according to the manufacturer’s instruction. The cytokine levels were detected by BD FACS CaliburTM flow cytometry (BD), and the results were analyzed by FCAP Array v3 software (BD).

### 2.9 Immunomodulatory effect of THP on CTX immunosuppressed mice

#### 2.9.1 Calculation of immune organs indices

The liver, spleen and thymus were obtained and weighed after the last administration. Organ index (%) = organ weight (g)/mouse weight (g) × 100.Blood was collected in EDTA-K2 contained centrifugal tubes and determined the content of HGB, WBC, RBC, PLT, LYMPH and NEUT by Coulter LH 755 Hematology Analyzer. The levels of C3 and C4 in serum were measured by ELISA kit (Nanjing jiancheng, China) according to the manufacturer’s instruction.

Carbon clearance test was used to detect the phagocytosis function of monocyte macrophages. Briefly, Each mouse was injected with 20% Indian ink at 10 mL/kg through the tail vein at 1 h after the last gavage.Blood samples were collected every 2 min (t1) and 10 min (t2), and a 20 μL sample was mixed with 0.1% sodium carbonate solution (2 mL). The optical densities D(λ) 1 and D(λ) 2 of t1 and t2 were measured at 600 nm by spectrophotometry. The carbon clearance index K was calculated The phagocytic index was calculated as follows: K = (lgOD1-lgOD2)/(t1-t2), where OD1 was for t1 and OD2 was for t2; phagocytosis index α = A/(B + C)×K ^1/3^, where A is the body weight of mice, B is the liver weight, and C is the spleen weight.

The spleens were washed with cold PBS, ground with a sterile glass rod and extruded through a 200-mesh sieve. The suspension was centrifuged at 1500 r/min for 3 min. The supernatant was discarded, and the precipitate was mixed with erythrocyte lysate. After standing for 5 min, the precipitate was centrifuged at 1500 r/min for 3 min and the supernatant was discarded. The centrifugation operation was repeated until the precipitate was white. The centrifugal liquid was suspended in RPMI 1640 medium containing 10% fetal bovine serum. Then, the cells were counted by microscopy, and the cell concentration was adjusted to 5×10^6^ cells/mL. The splenic cell suspension was inoculated into 96-well cell culture plates. The RPMI 1640 medium was added to the control group, and ConA (final concentration of 5 μg/mL) and LPS (final concentration of 10 μg/L) were added to the stimulation group. The cells were incubated in a 5% CO_2_ incubator at 37 °C for 48 h, the MTT method was used for detection. Stimulation index(SI) = OD_stimulation_/OD_control_.

Spleen lymphocyte suspension (1×10^7^ cells/mL, 100 μL), 1 μL CD4^+^ and 2.5 μL CD8^+^ monoclonal antibodies were added to the flow EP tube. The tube was blended and placed at 4 °C in the dark place for 15-30 minutes. At the same time, blank wells, CD4^+^ single staining wells, and CD8^+^ single staining wells were set. After incubation, 1 mL PBS was added to resuspend and the sample was centrifuged at 1500 rpm for 5 minutes. The supernatant was discarded, and 100 μL PBS was added to resuspend the cells, which was determined by flow cytometry. The percentage of T-lymphocytes and the CD4^+^/CD8^+^ ratio were calculated using the FCAP Array v3 software.

#### 2.9.2 Measurement of NK killing activity

Simultaneously, a mouse spleen lymphocyte suspension was used as effector cells. YAC-1 (8×10^4^ cells/mL) cells were used as target cells to determine the NK activity of the spleen. Splenocytes and YAC-1 cells were co-cultured in 96-well plates with an effector-to-target cell ratio of 25:1. After incubation for 4 h in a 5% CO_2_ atmosphere, the MTT method was used for detection. The killing activity of NK = [1- (effect-target cell OD value - effects OD value)/target cell OD value]×100%.

The liver, spleen and thymus tissues were fixed in 4% formaldehyde solution for more than 48 h, dehydrated with graded alcohol, and embedded in paraffin, then cut into 4 μm thick sections and stained with hematoxylin/eosin (H&E). Images were captured under a microscope (Eclipse TS100; Nikon Corporation, Tokyo, Japan).

### 2.10 Statistical analysis

The data were expressed as the mean ± standard deviation (SD) and compared by one-way analysis of variance (ANOVA), followed by LSD multiple comparisons test; ^*^p< 0.05, ^**^p< 0.01 and ^***^p< 0.001 were considered to be statistically significant. Histograms were generated using GraphPad Prism 8 (GraphPad Prism Inc., La Jolla, CA, USA). All data were analyzed with SPSS 25.0 statistical software.

## 3 Results

### 3.1 Chemical characterization and cytotoxic activity of THP

As shown in **Figure S1A, B**, a strong peak was found in the fractions eluted with 0.2 mol/L sodium chloride solution, which was collected for further purifification, while the fraction eluted with 0.5 mol/L sodium chloride solution presented a relatively weak peak. The molecular weight of THP determined by HPGPC was 66.2 kDa (**Supplemental Figure S1C**), and the total sugar and uronic acid contents of THP were determined as 83.3% and 48.9%, respectively. Furthermore, the monosaccharide composition of THP was determined using GC-MS after being hydrolysed and derivatised. The uronic acid in THP was mainly galacturonic acid (GalA) and the polysaccharide was mainly composed of GalA, glucose (Glc), mannose (Man), arabinose (Ara), galactose (Gal) and rhamnose (Rha) with molar ratios of 11.3:7.1:2.5:1.0:0.9:0.5 (**Supplemental Figure S1D**). The FT-IR spectrum of THP is shown in **Figure S1E**. The peaks at 3398.46 cm^-1^ and 2945.73 cm^-1^ indicated the O–H stretching of carbohydrates and C–H stretching of the aromatic structures and the methyl group, respectively. A absorption at 1618.34 cm^−1^ indicated the presence of carboxyl groups in THP, and the peak at 1745.16 cm^−1^ suggested the presence of uronic acid. The results of single-factor (**Figure S1F**) and response surface methodology (**Figure S1G**) experiments showed that the optimum conditions of extraction of THP were 2.25% of dosage of cellulase, 62.57 min of extraction time, 400.25 W of ultrasonic extraction power, and 29.72 mL/g of water to material ratio.

The result of MTT showed that THP had no significant effect on the viability of RAW264.7 cells (**Figure S2A**) and Lewis cells (**Figure S2B**) within the concentration range of 0–1,000 μg/mL.

### 3.2 Inhibitory effect of THP on tumor growth

**Figure 2:**
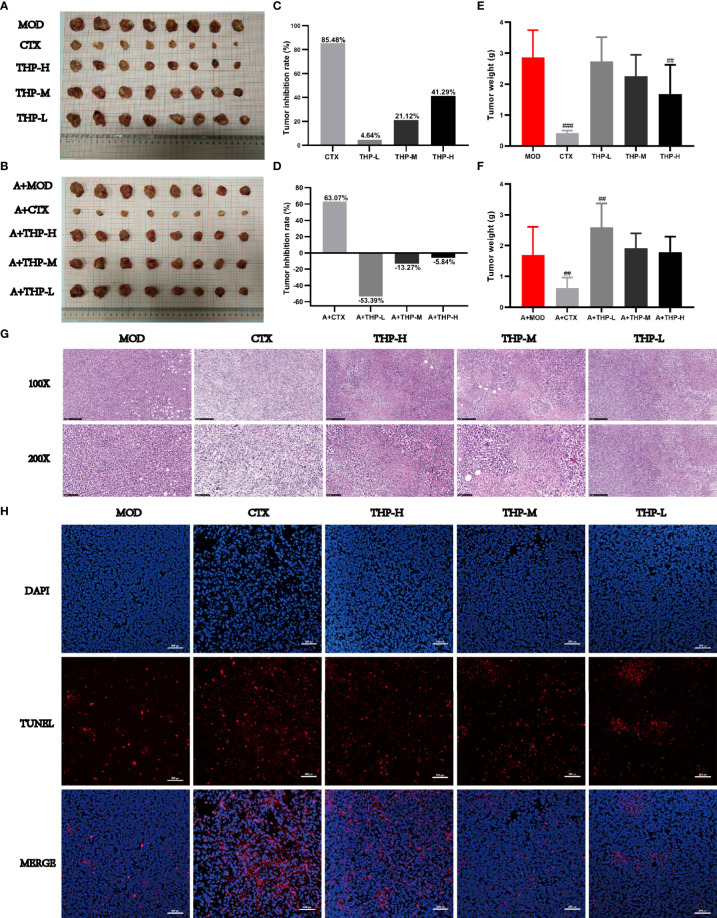
Effects of THP on tumor growth in Lewis tumor-bearing mice. **(A, B)** Photos of tumors. **(C, D)** Tumor inhibition rate. **(E, F)** Tumor index. **(G)** Histology of lung tumors. **(H)** Apoptosis of tumor cells. Each value is presented as the mean±SD. ^##^P<0.01, ^###^P<0.001 versus MOD.

As shown in **Figure 2**, the tumor volumes of the mice treated with CTX and THP after inoculation were smaller than those of the control group. In different THP treatment groups, the higher dose of THP showed a more significant effect in reducing the tumor volume, while the CTX group exhibited the most significant inhibition among all groups. By the end of the experimental period, the inhibition rate of Lewis tumors was 41.29% in the group treated with 200 mg/kg of THP, which showed a very significant differences compared with the CON group. The highest tumor inhibition rate among all groups of 85.47% was observed in the CTX group. However, there was no significant difference in tumor weight or tumor inhibition rate between the antibiotic+THP groups and the MOD group.

Histological changes in tumor tissues were observed *via* H&E staining. As shown in **Figure 2G**, tumor cells from MOD mice had an intact morphology with almost no necrotic cells, indicating normal growth of tumor cells. The tumor cells treated with CTX or THP showed different degrees of injury and apoptosis, and the number of normal tumor cells decreased. And microscopically, tumor cells in MOD group showed almost no apoptosis(**Figure 2H**). After CTX treatment, the number of apoptotic cells was significantly increased. Compared with the MOD group, the number of apoptotic cells increased significantly with the increase of THP concentration, which had a concentration-dependent apoptotic effect.

### 3.3 THP attenuated gut dysbiosis in Lewis tumor-bearing mice

**Figure 3:**
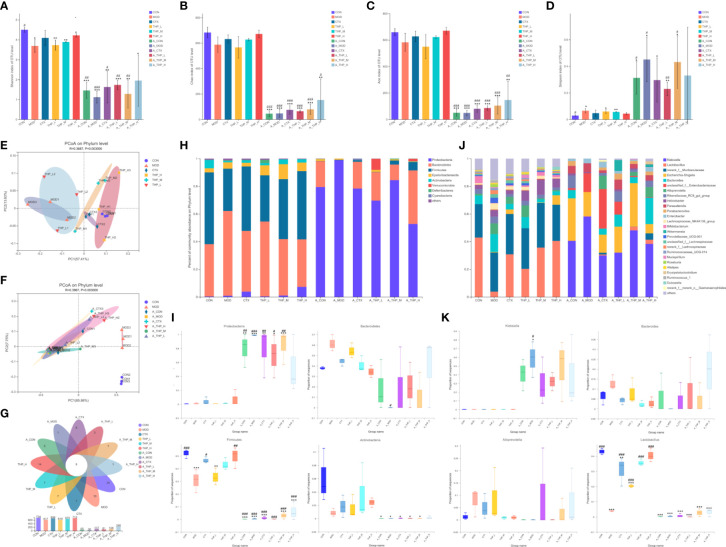
THP alters the abundance and diversity of intestinal flora. **(A)** Simpson indices. **(B)** Ace indices. **(C)** Chao indices. **(D)** Shannon indices. **(E, F)** PCoA at the phylum level. **(G)** Venn diagram. **(H)** Relative abundance of microbiota at the phylum level. **(I)** Characteristic bacteria at the phylum level. **(J)** Relative abundance of microbiota at the genus level. **(K)** Characteristic bacteria at the genus level. Each value is presented as the mean ± SD. ^*^P < 0.05, ^**^P < 0.01, ^***^P < 0.001 versus CON; ^#^P < 0.05, ^##^P < 0.01, ^###^P < 0.001 versus MOD.

Different alpha diversity indices were used to analyze gut microbial diversity and richness (**Figures 3A-D**). The Simpson, ACE, Chao and Shannon index results showed disordered intestinal microflora and significantly decreased intestinal microflora abundance in the antibiotic administration group. The THP high dose group (200 mg/kg) had a higher alpha diversity than the MOD group (ACE index [P = 0.105], Chao index [P = 0.097], Shannon index [P = 0.050]), suggesting that the THP treatment improved the richness of intestinal flora in mice. Common microbial β-diversity was further assessed by principal coordinate analysis (PCoA). PCoA based on Bray-Curtis distance, and showed clear differences between the MOD and THP groups (R = 0.3687, P = 0.003) and between the CON and antibiotic administration groups (R = 0.3987, P = 0.003), indicating that both THP and antibiotic treatment significantly affected microbial composition (**Figure 3E, F**). Furthermore, the proportion of shared and unique OTUs in the intestinal flora of each group was analyzed using plotting Venn plots (**Figure 3G**). There were 8 shared OTUs among the 12 groups. The numbers of unique OTUs in the CON, MOD, CTX, THP-L, THP-M, THP-H, A-CON, A-MOD, A-CTX, A-THP-L, A-THP-M and A-THP-H groups were 20, 10, 7, 7, 7, 14, 5, 1, 8, 3, 1 and 1, respectively.

To further clarify the taxa modulated by THP, the composition of the gut flora at the phylum level is shown in **Figures 3H, I**. After intestinal disorder induced by antibiotics, the abundance of p_Proteobacteria increased (P< 0.001), while the abundance of p_Bacteroidetes, p_Firmicutes and p_Actinobacteria decreased (P< 0.001). The abundance of Bacteroidetes in the CON group was 37.8% which was lower than that in the MOD group (61.33%). The abundances of Firmicutes and Actinobacteria in the CON group were 51.9% and 6.07%, respectively, which were reduced to 30.56% and 0.86% respectively, in the MOD group. THP treatment significantly increased Firmicutes and Actinobacteria (P< 0.01), but decreased Bacteroidetes compared with the MOD group. Analysis of the composition of bacteria at the genus level was shown in **Figures 3J, K**. After antibiotic induced intestinal flora disturbance in mice, as the abundance of g_Klebsiella, g_Parasutterella and g_Enterobacter showed an increasing trend (P< 0.001), the abundance of g_Lactobacillus in each group was greatly reduced (P< 0.001). Interestingly, the relative abundance of Lactobacillus was significantly increased (P< 0.001) and that of Alloprevotella and Bacteroides was decreased in the THP treatment groups compared with the MOD group. Therefore, the changes in the genus mentioned might be the key to the antitumor effects of THP.

**Figure 4:**
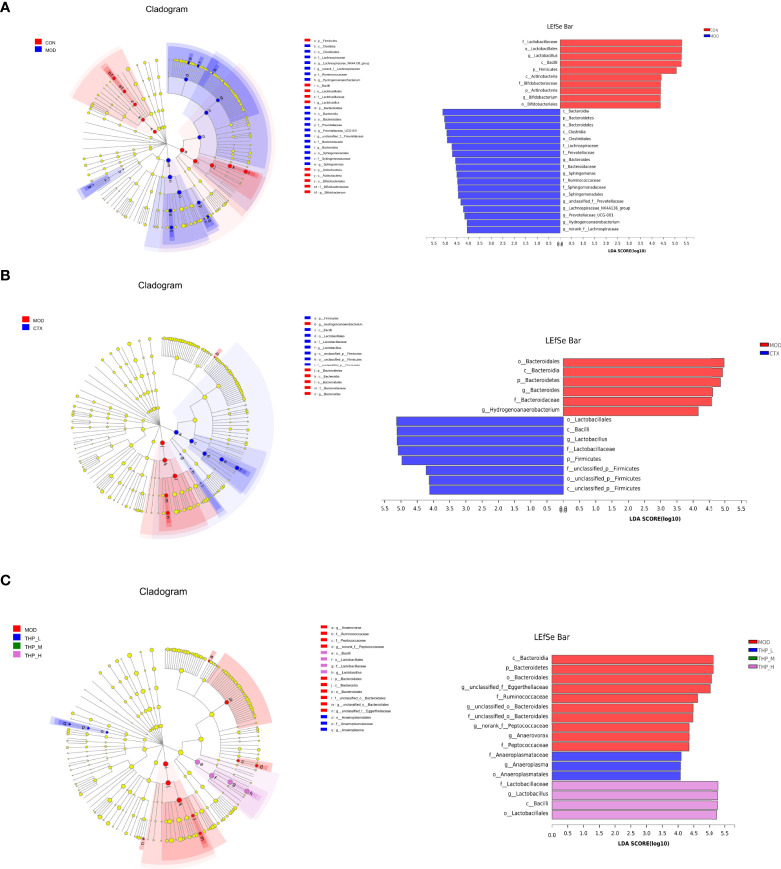
LEfSe comparison of intestinal microbiota. **(A)** CON group and MOD group. **(B)** CTX group and MOD groups. **(C)** MOD, THP-L, THP-M and THP-H groups.

Linear discriminate analysis (LDA) and effect size measurements (LEfSe) were used to further identify the key of microbial taxa among the different groups (**Figure 4**). According to the analysis results, Lactobacillaceae, Bacilli, Firmicutes, Actinobacteria, Bifidobacteriaceae, Actinobacteria, and Bifidobacterium displayed high LDA scores, showing that they were abundant in the CON group (LDA > 4). Compared with the CON group of mice, Bacteroidia, Clostridia, Lachnospiraceae, Prevotellaceae, Bacteroides, Sphingomonas, Ruminococcaceae, Sphingomonadaceae, and Hydrogenoanaerobacterium were enriched in the MOD group (LDA > 4). The results showed that Lactobacillales, Bacilli, and Firmicutes were significantly different in the CTX group compared to the MOD group(LDA > 4). In the THP-L and THP-H groups, Anaeroplasmataceae, Lactobacillaceae, and Bacilli exhibited higher scores, and the resulting composition was similar to that of the CON group, indicating that they were significantly affected by THP (LDA > 4). However, there were no dominant bacteria in THP-M according to the analysis (LDA > 4).

### 3.4 Effects of THP on the SCFAs content

**Figure 5:**
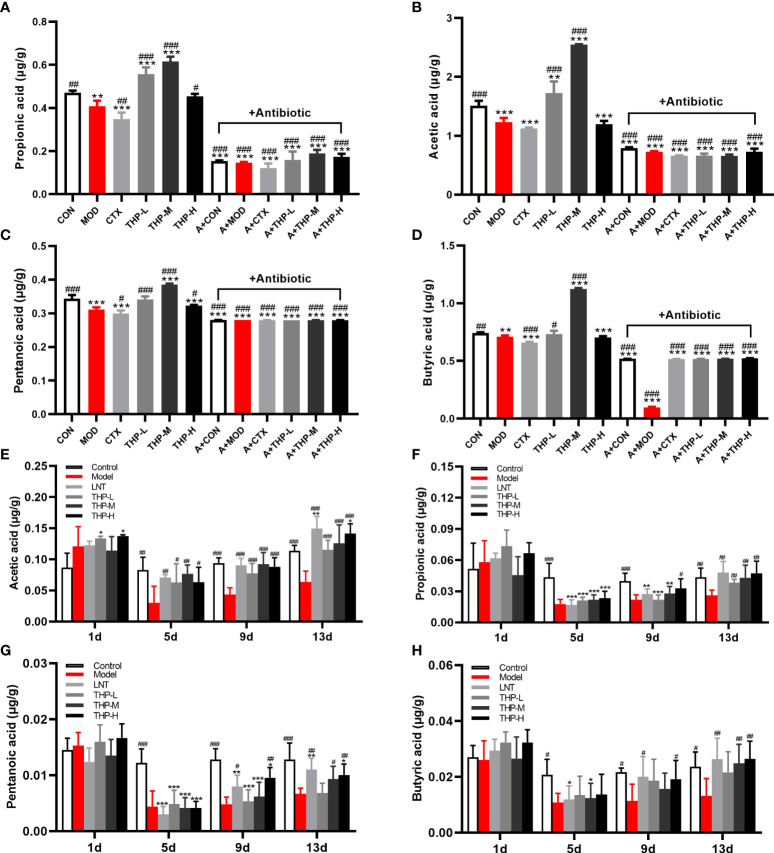
Effect of THP on the SCFAs content. **(A)** Propionic acid in Lewis tumor-bearing mice. **(B)** Acetic acid in Lewis tumor-bearing mice. **(C)** Pentanoic acid in Lewis tumor-bearing mice. **(D)** Butyric acid in Lewis tumor-bearing mice. **(E)** Propionic acid in immunosuppressed mice. **(F)** Acetic acid in immunosuppressed mice. **(G)** Pentanoic acid in immunosuppressed mice. **(H)** Butyric acid in immunosuppressed mice. Each value is presented as the mean ± SD. ^*^P < 0.05, ^**^P < 0.01, ^***^P < 0.001 versus CON or Control; ^#^P < 0.05, ^##^P < 0.01, ^###^P < 0.001 versus MOD or Model.

As shown in **Figures 5A-D**, after THP treatment, the contents of SCFAs showed an increasing trend (P< 0.05, P< 0.01, P< 0.001), and the increasing trend was most obvious in the THP medium-dose group. The contents of acetic acid, propionic acid, butyric acid and pentanoate acid increased to 2.55, 0.61, 1.12 and 0.39 in the THP-M group, respectively. Compared with the MOD group, the concentrations of SCFAs were significantly reduced after antibiotic treatment (P< 0.001). In particular, the content of SCFAs decreased most obviously after the combination of antibiotics and CTX. Surprisingly, the SCFA levels after CTX treatment were lower than those in the MOD group.

Therefore, the CTX model was used for further verification (**Figures 5E-H**). After adaptive feeding, there was no significant difference in the content of SCFAs in the feces of all groups. On Day 5, the SCFA levels in the Model, LNT, THP-L, THP-M and THP-H groups decreased significantly after CTX treatment (P< 0.05, P< 0.01, P< 0.001), indicating that the immunosuppressive model was successfully established. After 6 days of treatment (day 9), the SCFA content were significantly increased in the THP group (P< 0.05, P< 0.01, P< 0.001) and in the LNT group (P< 0.05, P< 0.001). On day 13, compared with the Control, the SCFA content did not increase significantly in the model group, but increased significantly in the LNT and THP treatment groups (P< 0.05, P< 0.01, P< 0.001). In the THP treatment group, the THP-H group had the best effect, and the contents of acetic acid, propionic acid, butyric acid and valeric acid content increased to 0.141, 0.047, 0.026 and 0.01, respectively.

### 3.5 Effects of THP on the secretion of SIgA in the intestinal tract

**Figure 6:**
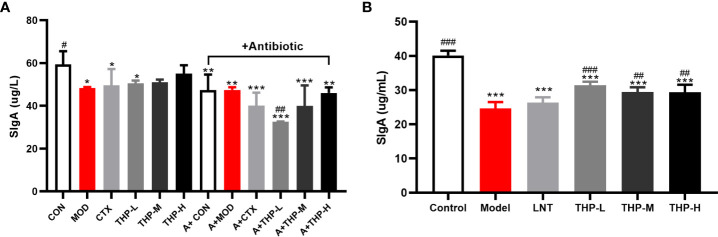
Effect of THP on the secretion of SigA in the intestinal tract. **(A)** SigA in Lewis tumor-bearing mice. **(B)** SigA in immunosuppressed mice. Each value is presented as the mean ± SD. ^*^P < 0.05, ^**^P < 0.01, ^***^P < 0.001 versus Control; ^#^P < 0.05, ^##^P < 0.01, ^###^P < 0.001 versus Model.

As shown in **Figure 6A**, the levels of SIgA in the antibiotic groups were significantly decreased, and the concentration of SIgA was at a low value of 32.55 μg/L in the A+THP-L group, while it showed an increasing trend with the increase of THP treatment groups. Compared with the MOD group, the SIgA content in the THP treatment group was significantly increased, with the most significant increase in the THP high-dose group (55.04 μg/L), while it was also found that the content of SIgA decreased in the CTX group. **Figure 6B** shows that the SIgA concentration in the Model group was significantly lower than that in the Control group (P< 0.001), while the SIgA concentration in the mice treated with LNT and THP was significantly higher than that in the Model group, especially THP-L (P< 0.001), suggesting that CTX inhibits intestinal mucosal secretion of SIgA, and THP can alleviate this situation. The effect of THP is better than that of LNT.

### 3.6 Effects of THP on body weight and organ index

**Figure 7:**
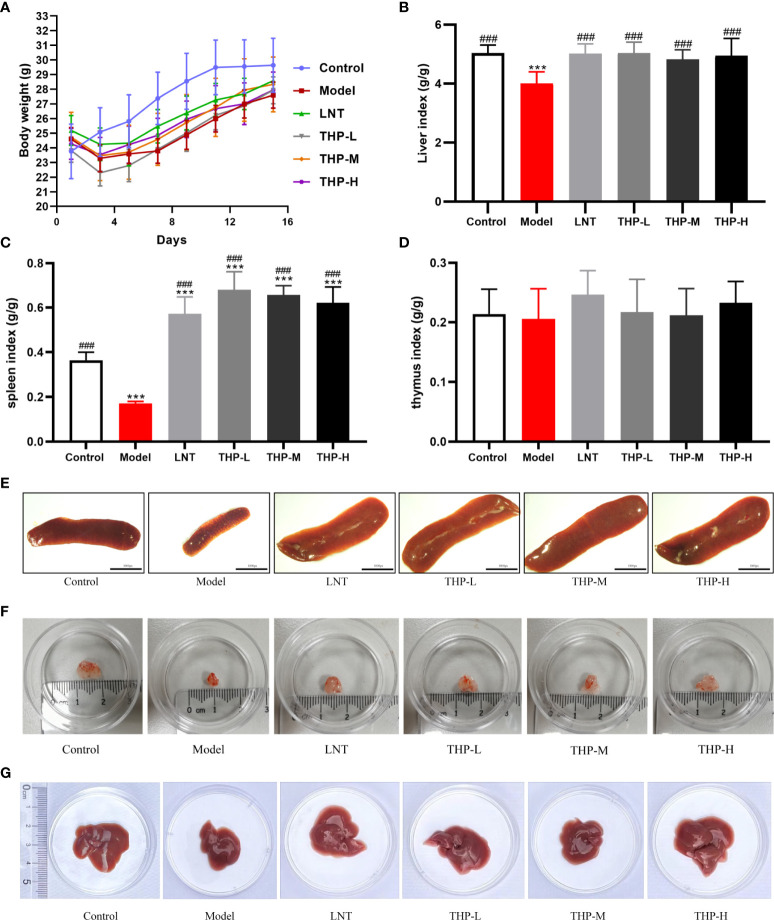
Effect of THP on the body weight and organ index. **(A)** Effect of THP on body weight. **(B)** Effect of THP on Liver index. **(C)** Effect of THP on the spleen index. **(D)** Effect of THP on the thymus index. **(E)** Effect of THP on the spleen. **(F)** Effect of THP on the thymus. **(G)** Effect of THP on the liver. Each value is presented as the mean±SD. ***P<0.001 versus Control; ^###^P<0.001 versus Model.

As shown in **Figure 7**, during the experiment, the body weight of the control group increased significantly, and the body weight of the model group first decreased significantly and then increased slowly after natural recovery. Body weight recovered significantly when mice were treated with THP and LNT. The increase was the most obvious in the THP low-dose group, with an increase of 4.12 g, which was significantly different. The liver index, thymus index and spleen index were measured to observe the effect of THP on CTX-treated mice. The experimental results showed that the liver and spleen indices of the model group were significantly decreased compared with those of the control group (P< 0.001), to 4.01 and 0.17, indicating that the immunosuppressive model was successfully established. Compared with the model group, the liver and spleen indices of LNT group and THP treatment group were significantly increased (P< 0.001). In particular, the THP low-dose group had the best effect on improving the organ index of mice (the indices of liver and spleen increased by 5.04 and 0.68, respectively), and the effect was better than that of the LNT group.

### 3.7 Effects of THP on the blood index and cytokine content

**Figure 8:**
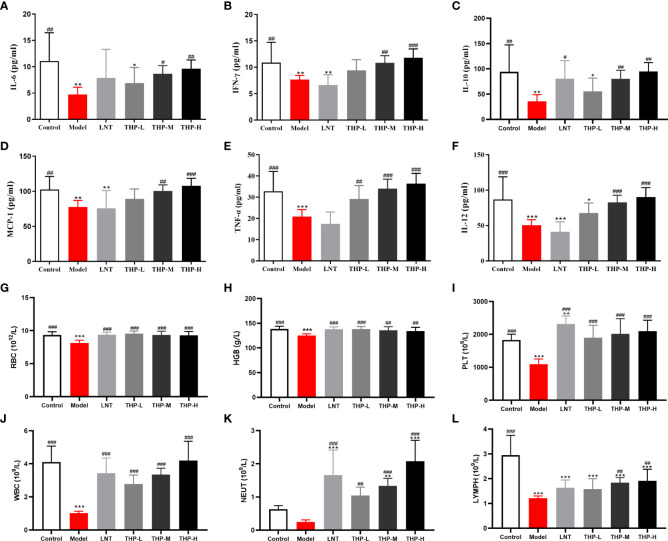
Effect of THP on CTX-induced immunosuppression through humoral immunity. **(A)** IL-6. **(B)** IFN-γ. **(C)** IL-10. **(D)** MCP-1. **(E)** TNF-α. **(F)** IL-12. **(G)** RBC. **(H)** HGB. **(I)** PLT. **(J)** WBC. **(K)** NEUT. **(L)** LYMPH. Each value is presented as the mean ± SD. ^*^P < 0.05, ^**^P < 0.01, ^***^P < 0.001 versus Control; ^#^P < 0.05, ^##^P < 0.01, ^###^P < 0.001 versus Model.

As shown in **Supplemental Figure S3**, in lewis tumor-bearing mice, compared with the CON group, the concentrations of IL-2, TNF-α and INF-γ in the MOD group were decreased (P< 0.001). There was a significant rise in the IL-2, TNF-α, and INF-γ concentrations after THP treatment (P< 0.01, P< 0.001). The concentrations of IL-2, TNF-α and INF-γ increased from 44.914pg/ml, 133.519pg/ml and 143.3pg/ml to 47.431pg/ml, 162.185pg/ml and 164.6pg/ml, respectively in THP-H group. But surprisingly, the concentrations of IL-2 and INF-γ have tended to decrease instead after treatment with CTX. As shown in **Figures 8A-F**, the serum levels of IFN-γ, IL-10, TNF-α, IL-12, IL-6 and MCP-1 in the Model group were lower than those in the Control group (P< 0.01, P< 0.001). At doses of 100 mg/kg and 200 mg/kg, the levels of IFN-γ, IL-10, TNF-α, IL-12, IL-6 and MCP-1 were significantly increased (P< 0.05, P< 0.01, P< 0.001). Especially at a dose of 200 mg/kg, the levels of IFN-γ, IL-10, TNF-α, IL-12, IL-6 and MCP-1 were restored to or even above the normal level, demonstrating that THP could improve the cytokine content in CTX-treated mice.

As shown in **Figures 8G-L**, the levels of WBC, RBC, HGB, PLT and LYMPH in mice were significantly reduced after CTX treatment (P< 0.001). After treatment, these levels improved significantly in the mice treated with LNT and THP compared to the model group (LNT group, P< 0.001; THP group, P< 0.01, P< 0.001). In particular, the WBC, LYMPH and NEUT counts of the 200 mg/kg THP group were higher than those of the LNT group. Moreover, the effects of THP on PLTs, WBCs, NEUTs and LYMPHs were dose-dependent.

### 3.8 Effects of THP on the immunoglobulin and complement

The changes inimmunoglobulin concentrations in serum are shown in **Supplemental Figure S3**. The concentrations of IgG, IgA and IgM in the MOD group were lower than those of the CON group as expected (P< 0.05, P< 0.001). Compared with the MOD group, the contents of IgG and IgM after THP treatment were significantly increased (P< 0.01, P< 0.001), except that the IgA content after THP-H treatment showed a downward trend. The concentration of immunoglobulin decreased after CTX treatment. As shown in **Table 1**, the levels of IgM, IgG and IgA in the Model group were lower than those in the Control group. Compared with the model group, the levels of serum total IgA and IgM were significantly increased in the THP treatment group (P< 0.01, P< 0.001). The IgG level of the THP high dose group increased most significantly (P< 0.01), and the IgG level of the THP treatment group was higher than that of the control group. To further explore the effect of THP on humoral immunity in CTX-immunosuppressed mice, the serum levels of C3 and C4 in mice were investigated (**Table 1**).The expression of C3 in the model group was lower than that in the Control group. After treatment with THP, the levels of C3 and C4 increased, especially in the THP-L group.

**Table 1:** Effects of THP on immunoglobulin and complement in mice.

Group	IgM (mg/mL)	IgA (mg/mL)	IgG (mg/mL)	C3 (mg/mL)	C4 (mg/mL)
Control	1110.651±183.908	2.016±0.363	11.523±1.281	1133.139±116.879	138.798±31.295
Model	1077.173±61.206	1.843±0.321	9.648±4.246	941.215±35.845	192.034±32.883
LNT	1112.322±87.244	2.447±0.387 ^#^	14.937±1.425	849.822±73.840	210.406±14.243^*^
THP-H	1372.634±124.477	2.580±0.446^* ##^	19.865±3.267^* ##^	817.653±161.842	202.566±52.218^*^
THP-M	1695.445±240.755^**##^	3.101±0.367 ^*** ###^	15.524±4.413^#^	1272.98±282.595	200.102±30.675^*^
THP-L	1658.176±227.236^**##^	3.163±0.530 ^*** ###^	10.741±4.921	1106.750±125.733	221.973±22.218^**^

Each value is presented as mean±SD. ^*^P < 0.05, ^**^P < 0.01, ^***^P < 0.001 versus Control; ^#^P < 0.05, ^##^P < 0.01, ^###^P < 0.001 versus Model.

### 3.9 Effects of THP on the splenic lymphocytes, NK-cell activity and macrophage phagocytic activity

**Figure 9:**
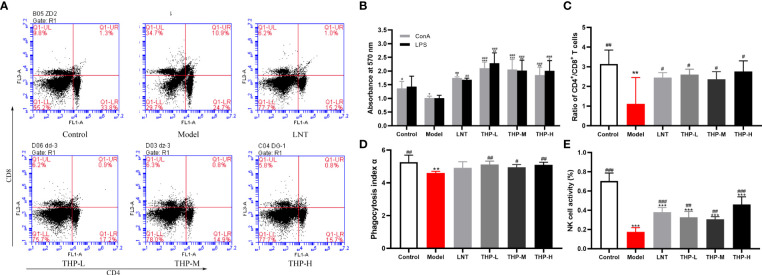
Effect of THP on CTX-induced immunosuppression through cellular immunity. **(A)** The proportion of splenic CD4^+^CD8^+^ T cells was detected by flow cytometry. **(B)** The ratio of splenic CD4^+^/CD8^+^ T cells. **(C)**Lymphocyte proliferation. **(D)** Phagocytic index α. **(E)** NK-cell activity. Each value is presented as the mean ± SD. ^*^P < 0.05, ^**^P < 0.01, ^***^P < 0.001 versus Control; ^#^P < 0.05, ^##^P < 0.01, ^###^P < 0.001 versus Model.

It is shown in **Figure 9B** that CTX significantly decreased the proliferation of splenocytes in the model group compared with the control group (P< 0.05). Supplementation with THP and LTN reversed this decline and accelerated the differentiation of T and B lymphocytes, and the effect of THP was particularly obvious. In addition, the proliferation rates in the THP low-dose group and THP high-dose group were significantly higher than that in the LNT treatment group. At a dose of 50 mg/kg, the proliferation effect of lymphocytes was most obvious (P< 0.001). As shown in **Figures 9A, C**, compared with the control group, treatment with CTX significantly reduced the proportion of CD4^+^ T lymphocytes and the ratio of CD4^+^ to CD8^+^ cell and significantly increased the proportion of CD8^+^ T lymphocytes (P< 0.01). The cellular immune function of mice was inhibited, indicating that the immunosuppressed mouse model was successfully constructed. The CD4^+^/CD8^+^ ratio in the THP-L and THP-H groups significantly increased (P< 0.05), from 1.12 to 2.76 and 2.60, respectively, and the effect was better than that in the LNT group.

The phagocytic function of mononuclear macrophages in mice was measured by a carbon clearance test (**Figure 9D**). A significant decrease in the phagocytic index α of the model samples was observed compared with that of the control group (P< 0.01). Compared with the model group, the phagocytosis function of THP-treated mice was significantly improved (p< 0.05, P< 0.01). Especially at a dose of 50 mg/kg, phagocytosis function was restored to the normal level or even above the normal level (increased to 5.12), and the effect of THP was better than that of LNT. The activity of NK cells in the Model group treated with CTX was significantly lower than that in the control group, shown in **Figure 9E** (P< 0.001), indicating that the immunosuppression model was successfully established. Administration of THP showed a statistically significant (p< 0.01, P< 0.001) reverse in the decline in NK-cell.activity in immunosuppressive mice. The THP high-dose group showed the most significant therapeutic effect, which increased to 0.46, and the effect was better than that of LNT.

### 3.10 Effect of THP on pathological changes in liver tissue, spleen tissue and thymus tissue

**Figure 10:**
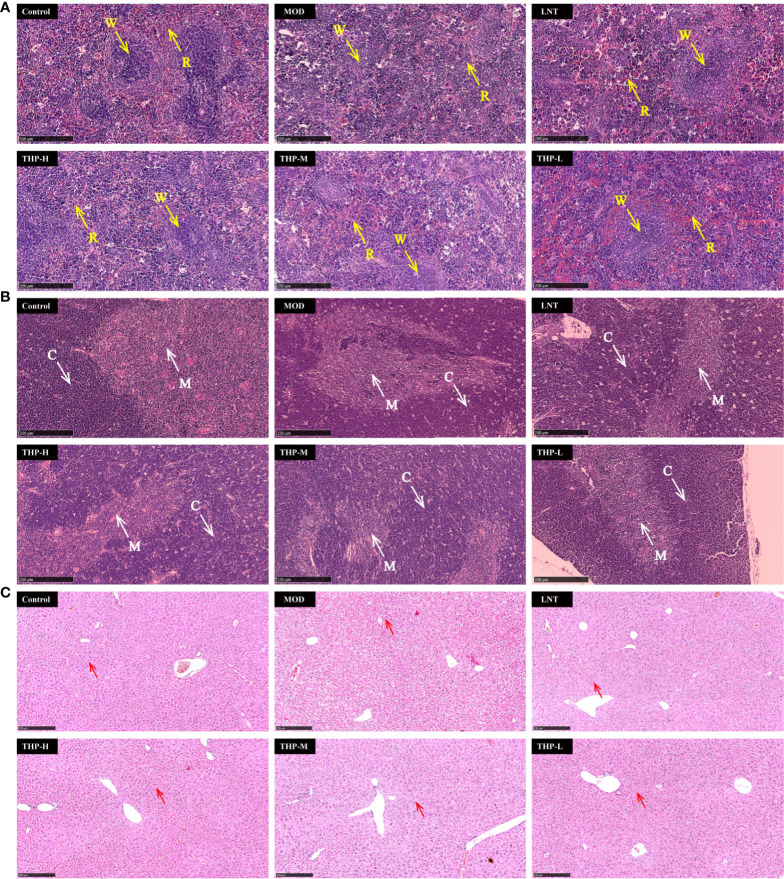
Effect of THP on the histopathology of the spleen, thymus and liver. (R, red pulp. W, white pulp. C, cortex. M, medulla. Red arrow: Kupffer cell) Scale bar: 250 μm. **(A)** HE staining of the spleen,**(B)** HE staining of the thymus, **(C)** HE staining of the liver.

As shown in **Figure 10A**, pathological changes in spleen tissues stained by HE showed that the boundary between the red pulp and white pulp of the spleen in the control group was clear, and the central splenic artery was visible. The boundary between the white pulp and red pulp in the Model group was blurred and the structure was disorganized, indicating a certain degree of necrosis in the splenic tissue. After treatment with LNT and THP, the pathological state of the splenic tissue was improved, the boundary between the white pulp and red pulp became clear, and the color of the white pulp became darker. As shown in **Figure 10B**, the thymus cortex and medulla of the control group were demarcated, with deep staining of the cortex. In the Model group, the cortical and medulla boundaries were blurred, the cortical color was light, and the tissue showed a high degree of necrosis. The boundaries between the cortex and medulla were clear, and the color of the cortex was darker in the LNT group, which was improved as a whole but not as complete and clear as the control group. After THP treatment, the pathological status of the thymus tissue was improved, and the best effect was achieved with a high dose. The boundary between the cortex and medulla was clear, and the cortex was deeply stained. The histological morphology of liver were shown in **Figure 10C**. In the control group, the cells were centered in the liver cord and arranged radially, with few intracellular vacuoles and large amount of Kupffer cells. Instead, the liver cells in the model group were disordered and contained a large number of vacuoles, with a significant reduction of Kupffer cells. LNT and THP effectively ameliorated the liver damage caused by CTX, significantly increased the Kupffer cells, and the structure was roughly radially arranged.

## 4 Discussion

With the continuous development and research of polysaccharides in TCM, more than 300 polysaccharide compounds have been extracted from natural products, and polysaccharides contained in many medicinal plants, such as Polygala tenuifolia Willd (43), Eriobotrya japonica (44) and Dendrobium (45) have been found to have antitumor effects. This study investigated a novel TCM with medicinal value. The experimental results confirm that THP inhibited both tumor size and body weight in Lewis tumor-bearing mice, indicating that THP has an antitumor effect. However, the experimental results also show that CTX had a better antitumor effect than THP.

The composition of the intestinal flora affects the effect of anticancer immunity. Patients for whom treatment is effective have a high diversity of flora, and specific strains, such as Bifidobacterium, Lactobacillusis and (46), and B fragilis (47). These bacteria have a positive effect on general health, reducing the incidence of metabolic disorders and a variety of chronic inflammatory diseases. Most cancer patients have intestinal microbiota disorders (48), there were fewer bacteria in the acetate- and butyrate-producing family, while Klebsiella and Haemophilus which produce LPS, were increased (49). Normal intestinal microbiota helps enhance the efficacy of anticancer drugs. In this study, a pseudo-sterile tumor-bearing mouse model was established using an antibiotic cocktail and Lewis lung cells and found that the antitumor effect of THP in intestinal disorder mice was poor, indicating that the antitumor effect of THP was closely related to intestinal flora. Based on the evaluation of the above experimental results, we further analyzed the changes in intestinal flora. The results of 16S rRNA sequencing experiments showed that THP could increase the abundance of Lactobacillus, and reduce the abundance of Bacteroides. It has been demonstrated that Lactobacillus are important probiotics for maintaining intestinal microbial homeostasis (50). Moreover, studies have proven that the extracellular polysaccharide produced by Lactobacillus can effectively inhibit the proliferation of colon cancer cells *in vitro* (51). High concentrations of enterotoxigenic Bacteroides fragilis in the genus Bacteroidetes are closely associated with colorectal cancer (52). We believe that the changes in these two intestinal microflora may be an important link for THP to exert its antitumor effects.

The intestinal flora is closely linked to the production of SCFAs (53). Studies have shown that increased dietary fiber intake is associated with anti-inflammatory and anticancer effects (54). This is related to the SCFAs produced by dietary fiber fermentation, which are easily absorbed and have positive systemic physiological effects on the host. Intestinal mucosal SIgA is an important effector molecule protecting mucosal immunity. It cooperates with other innate and adaptive immune mechanisms to maintain host immune homeostasis (55). In this study, after THP treatment, SIgA and SCFAs in Lewis tumor mice were improved, indicating that THP can promote the secretion of SIgA and SCFAs. This demonstrates that polysaccharides can exert antitumor effects by affecting intestinal mucosal immunity and the secretion of SCFAs. However, it was surprising to find that the SCFAs and SIgA content in CTX-treated mice were lower than those in the model. Therefore, a CTX model was established, and THP alleviated the decreased content of SCFAs and SIgA induced by CTX.

CTX is an effective tumor chemotherapy drug, that widely used in the treatment of various cancers and autoimmune diseases. However, it is limited in the application of clinical chemotherapy due to its numerous side effects, especially immunosuppression (56). Several studies have shown that many polysaccharides can alleviate CTX-induced immunosuppression (57, 58). Based on our above findings that the antitumor effect of CTX is superior to that of THP and THP improved the reduced content of SCFAs and SIgA caused by CTX, we considered whether THP could ameliorate the immunosuppressive side effects of CTX. Therefore, a CTX immunosuppressive mouse model was established. The results show that THP could ameliorate the immunosuppressive side effects of CTX through specific and nonspecific immunity.

Nonspecific immunity is the second barrier of defense against invading microbial pathogens and other potential threats and is essential for maintaining host defense and self-tolerance. The index of the liver, spleen and thymus can reflect the immune ability of the body and is an intuitive indicator of the strength of the innate immune function of the body (59). The decrease in the immune organ index is a typical symptoms of immunosuppressive mice, and the body weight (60). According to the observation of splenic histopathology, the model spleen showed mixed red and white pulp, indicating that the CTX-immunosuppressed mouse model was successfully established. And these symptoms improved significantly after treatment with THP. Furthermore, as expected, the organ index of the treatment group was improved, indicating that the immune activity of immunosuppressive mice was ameliorated.

To further understand the effect of THP on nonspecific immunity, we determined other nonspecific immunity-related indicators. Macrophages are important mediators in the nonspecific immune response (61). The phagocytosis of mononuclear macrophages is usually used to evaluate the nonspecific immune status of animals (62). The carbon clearance test can reflect the phagocytic capacity of mononuclear macrophages (63). In the experiment, we found that THP can restore the phagocytosis of mononuclear macrophages in immunosuppressive mice to a certain extent. Coincidentally, relevant studies have also shown that polysaccharides can promote the phagocytosis of monocyte macrophages and have certain immunomodulatory activity, which is consistent with our study results (64). NK-cell activity is also an important indicator of the nonspecific immune system (65). The results showed that THP can enhance the activity of NK cells to regulate the body’s immunity, and can be used as an effective therapy to improve the immunodeficiency. These findings are consistent with previous reports of significantly enhanced macrophage phagocytosis and NK-cell activity in immunocompromised mice treated with polysaccharides (66). These results indicate that THP plays an important role in regulating nonspecific immunity of the body and may be used as an effective drug to improve immunodeficiency.

Specific immunity is another branch of the body’s immune defense system in addition to specific immunity. A specific immune response can recognize and clear pathogens, protect the body from “nonself” substances and is an important line of defense of the body’s immunity. Specific immunity includes humoral immunity and cellular immunity, involving the effect mechanism of lymphocytes, cytokines, complement and antibodies (67). Lymphocytes are the key cells in the specific immune response (68). The proliferation ability and function of splenic lymphocytes is an important index to evaluate the immune ability of the body (69). ConA-induced cell proliferation is often used to detect T lymphocyte immunity, while B lymphocytes are more sensitive to LPS (70). Therefore, in this study, ConA and LPS were selected to induce the proliferation of T and B lymphocytes. To better understand the effects of THP on lymphocytes, we further studied the T lymphocyte subsets. T cells can be divided into two subsets, CD4^+^ T cells and CD8^+^ T cells, which are the central link between the body’s immune response and immune regulation (71). When the ratio of CD4^+^ to CD8^+^ is out of proportion or the function is changed, it can lead to the immune regulation dysfunction of the body and cause a series of pathological changes (72). Excitingly, our results showed that THP significantly improved spleen lymphocyte proliferation and CD4^+^/CD8^+^ ratio in CTX-induced immunosuppressed mice, suggesting that THP effectively regulated cellular immune function in cyclophosphamide immunosuppressed mice. The experimental results are consistent with those previously published for results induced by polysaccharides in this assay (73).

Humoral immunity plays a key role in the adaptive immune system activated by specific antigens (74). IgA, IgM and IgG are the major immunoglobulins involved in complement activation, ionization, and toxin neutralization by specific binding with pathogenic microorganisms to neutralize toxins and pathogens (75). An increasing number of studies have proven that polysaccharides isolated from Ganoderma atrum (76), Salvia miltiorrhiza (77), Hippophae rhamnoides (78) and other plants can enhance humoral immunity by promoting the secretion of specific IgA, IgM and IgG. IFN-γ is released by Th1, which activates macrophages and induces cellular immunity (79). IL-10 and IL-6, released by Th2 cells, induce the proliferation of B cells and mediate humoral immunity (80). Although TNF-α is well known for its proinflammatory activity, it also plays an important role in increasing immunity (81). IL-12 promotes T-bet expression and Th1 differentiation, which stimulates the secretion of IFN-γ and TNF-α (82). Decreased MCP-1 secretion is a key indicator of the impaired immune status of monocytes (83). Findings from earlier reports have suggested that some polysaccharides can improve immune suppression by upregulating the levels of cytokines. As we expected, in this study, serum IgM, IgG, IgA, IFN-γ, IL-10, TNF-α, IL-12, IL-6, MCP-1 and C3 levels in mice increased in immunosuppressed mice treated with THP, suggesting that THP can promote humoral immunity.

In addition, through routine blood routine examination, we can diagnose immune-related diseases according to the main components, distribution and cell morphology of blood. It has been reported that RBCs and HGB play important roles in the innate immune system, participating in the regulation of the immune response (84). The change of WBC count is an important indicator to diagnose cancer and immune diseases (85). Our study shows that THP can effectively normalize CTX-induced routine blood parameters, suggesting that THP can effectively improve the immunity of immunosuppressed mice.

In our previous studies, we found that THP enhances immunity and exhibits anticancer activity (26). Meanwhile, some scientists have proposed that the anti-tumor mechanism of *Tetrastigma hemsleyanum* may include calcium signaling pathway (86), PI3K/AKT/mTOR signaling pathway (87) and CDK6 and MET genes (88). We believe that the anti-tumor effect of TCM is multi-target and multi-mechanism. In this study, we further proved that the anticancer activity of THP may be related to intestinal flora and immunomodulatory effect.

## 5 Conclusion

This study established a clinically related murine Lewis lung cancer model and demonstrated that THP can regulate intestinal flora to play an antitumor role. In addition, we also confirmed that THP may enhance immune function in immunosuppressed mice by enhancing the immune organ index, and innate and adaptive immunity, and improving intestinal mucosal immunity. Overall, the results indicate that THP is an effective immunomodulator and antitumor drug, and the combination of THP with chemotherapy drugs can improve the antitumor effect, enhance immunoregulation, relieve immunosuppression stress, and reduce the toxicity of chemotherapy.

## Data availability statement

The datasets presented in this study can be found in online repositories. The names of the repository is jianguoyun and accession number is https://www.jianguoyun.com/p/DTz_09MQ4_rXChjZu8cEIAA.

## Ethics statement

This study was reviewed and approved by Experimental Animal Center of Zhejiang Chinese Medicine University the license number: SYXK(Zhe)2021-0012.

## Author contributions

ZD initiated and designed the study. FZ, YL, TS, LS, BW, JL, ZL and BZ performed the experiments. FZ and YL performed the statistical analysis and prepared the manuscript. SH and ZD edited and approved the manuscript. All authors contributed to the article and approved the submitted version.

## Funding

This research was supported by the National Natural Science Foundation of China (Grant No. 82141210); The university-level scientific research project of Zhejiang Chinese Medicine University (Grant No. 2022JKZKTS19).

## Acknowledgments

Thanks for the technical and experimental support of the Public Platform of Medical Research Center, Academy of Chinese Medical Science of Zhejiang Chinese Medicine University.

## Conflict of interest

The authors declare that the research was conducted in the absence of any commercial or financial relationships that could be construed as a potential conflict of interest.

## Publisher’s note

All claims expressed in this article are solely those of the authors and do not necessarily represent those of their affiliated organizations, or those of the publisher, the editors and the reviewers. Any product that may be evaluated in this article, or claim that may be made by its manufacturer, is not guaranteed or endorsed by the publisher.

## Supplementary material

Supplementary Figure 1 Effects of THP on viability of RAW 264.7 and Lewis cells. Each value is presented as the mean ± SD. **(A)** RAW264.7. **(B)** Lewis cells.

Supplementary Figure 2 Isolation and characterization of THP. **(A)** Anion-exchange chromatogram. **(B)** Gel chromatographic profile on Superdex-200 column. **(C)** HPGPC chromatogram of THP. **(D)** FT-IR spectrum of THP. **(E)**GC-MS total ion chromatogram: **(A)** Monosaccharides standards, **(B)** THP, and **(C)** Reduction product of THP. **(F)** The effect of the dosage of cellulase, extraction time, extraction power, and the ratio of water to material on the extraction rate of THP. **(G)** Response surface plots of extraction rate of THP

Supplementary Figure 3 Effect of THP on cytokine and immunoglobulin levels in the serum of Lewis tumor-bearing mice. Each value is presented as the mean ± SD. **(A)** IL-2. **(B)** TNF-α. **(C)** IFN-γ. **(D)** IgG. **(E)** IgM. **(F)** IgA.*P<0.05, **P<0.01, ***P<0.001 versus Control; ^#^P<0.05, ^##^P<0.01, ^###^P<0.001 versus Model.
