# Supplementary figures and images for: Antitumor effects of polysaccharides from Tetrastigma hemsleyanum Diels et Gilg via regulation of intestinal flora and enhancing immunomodulatory effects in vivo
Source: Front Immunol. 2022 Oct 28;13:1009530. doi: 10.3389/fimmu.2022.1009530 (PMC9650377; doi:10.3389/fimmu.2022.1009530)

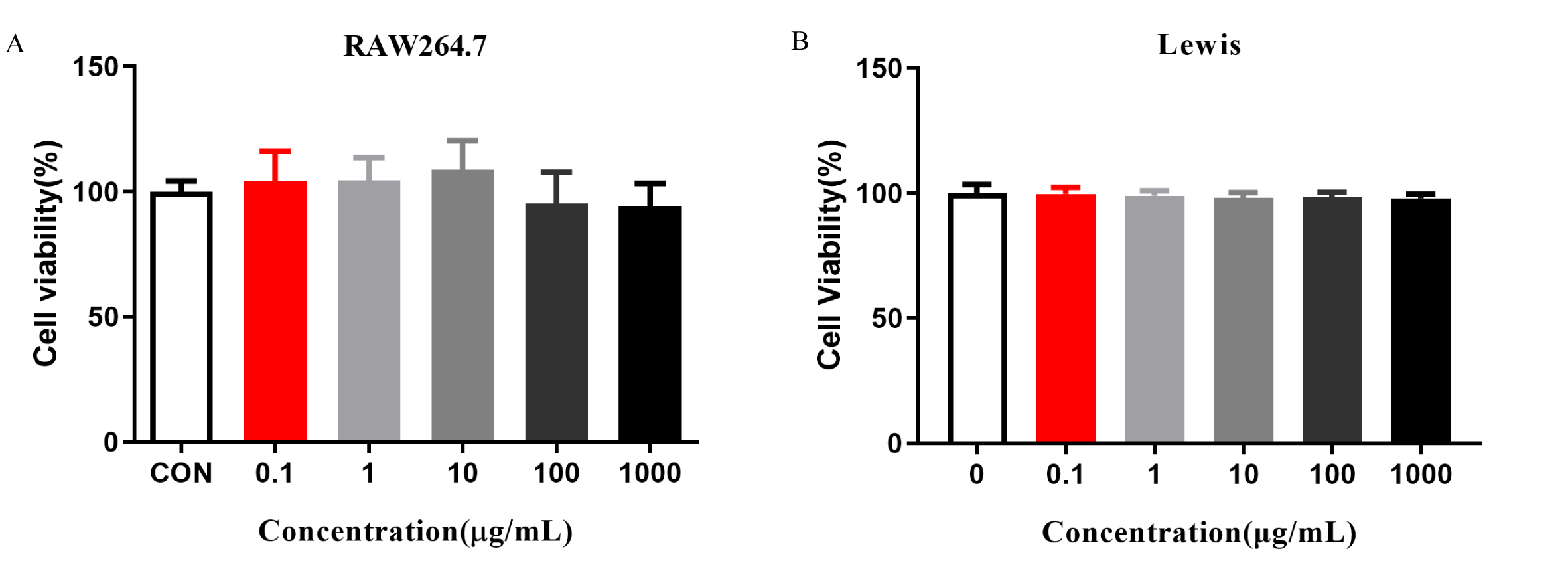

Supplement: Supplementary Figure 1 — Effects of THP on viability of RAW 264.7 and Lewis cells. Each value is presented as the mean ± SD. (A) RAW264.7. (B) Lewis cells. [file Image_1.tif]

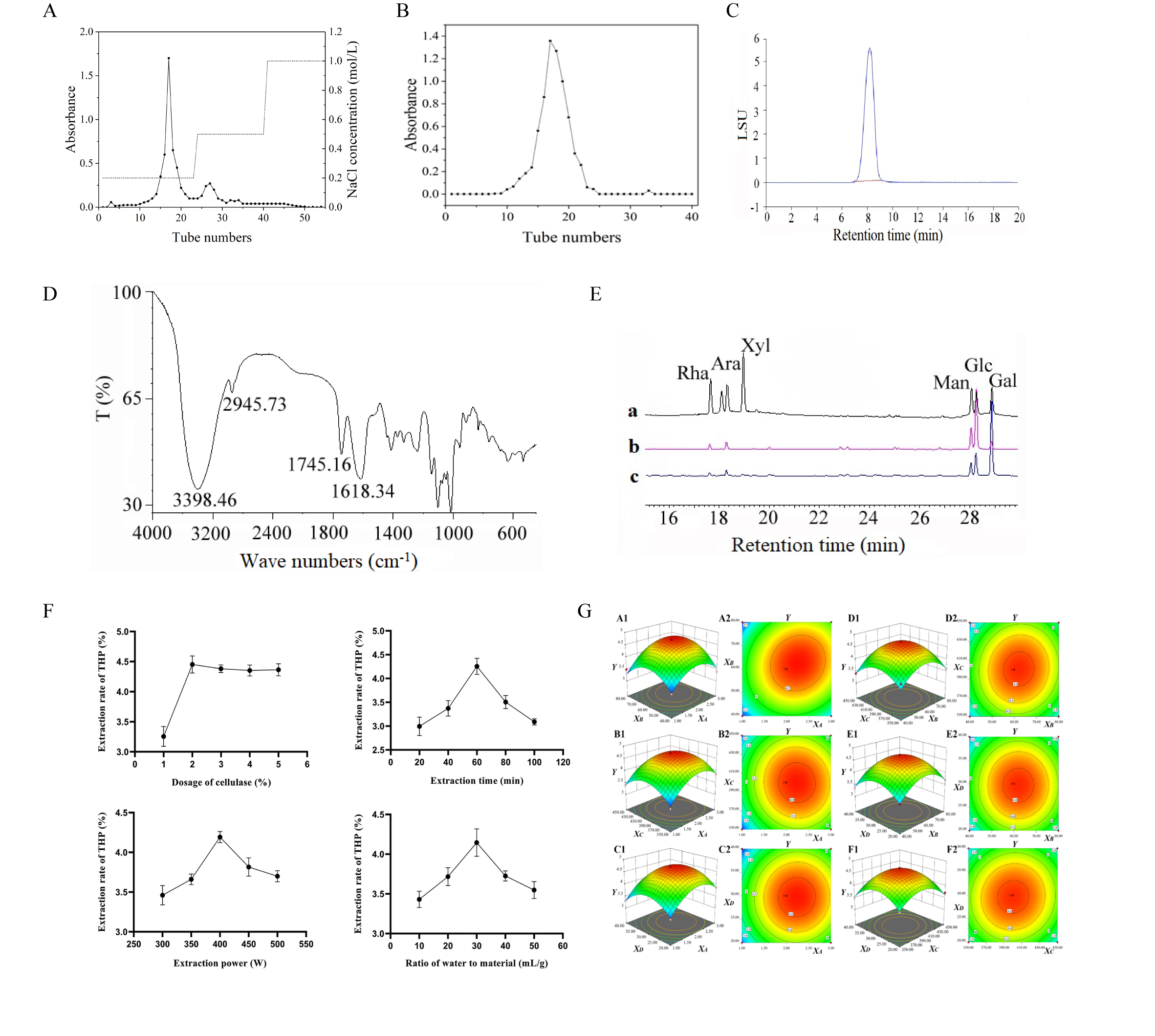

Supplement: Supplementary Figure 2 — Isolation and characterization of THP. (A) Anion-exchange chromatogram. (B) Gel chromatographic profile on Superdex-200 column. (C) HPGPC chromatogram of THP. (D) FT-IR spectrum of THP. (E)GC-MS total ion chromatogram: (A) Monosaccharides standards, (B) THP, and (C) Reduction product of THP. (F) The effect of the dosage of cellulase, extraction time, extraction power, and the ratio of water to material on the extraction rate of THP. (G) Response surface plots of extraction rate of THP [file Image_2.tif]

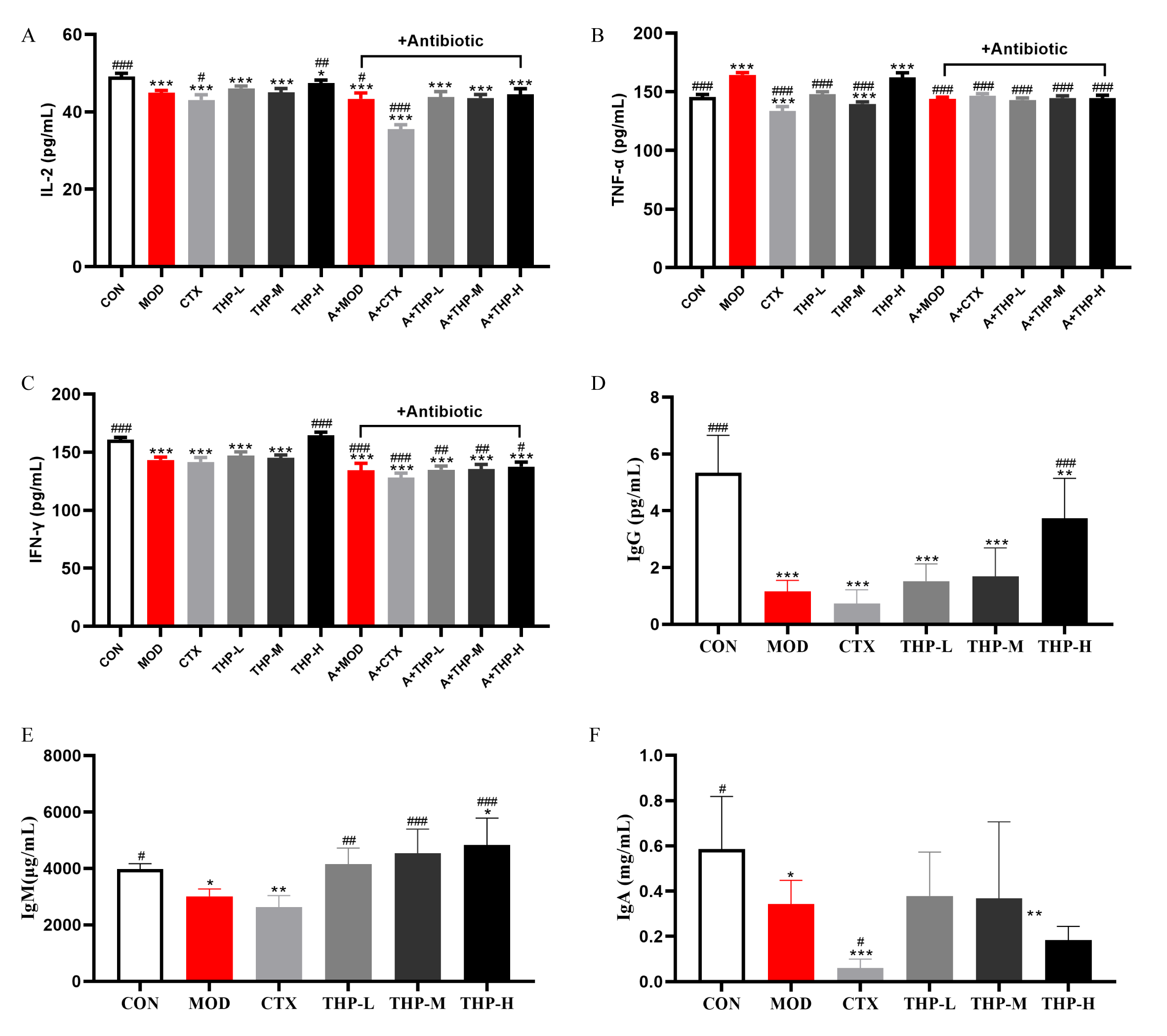

Supplement: Supplementary Figure 3 — Effect of THP on cytokine and immunoglobulin levels in the serum of Lewis tumor-bearing mice. Each value is presented as the mean ± SD. (A) IL-2. (B) TNF-α. (C) IFN-γ. (D) IgG. (E) IgM. (F) IgA.*P<0.05, **P<0.01, ***P<0.001 versus Control; #P<0.05, ##P<0.01, ###P<0.001 versus Model. [file Image_3.tif]
